# Methods for assessing cardiac myofilament calcium sensitivity

**DOI:** 10.3389/fphys.2023.1323768

**Published:** 2023-12-05

**Authors:** Jarrah M. Dowrick, Andrew J. Taberner, June-Chiew Han, Kenneth Tran

**Affiliations:** ^1^ Auckland Bioengineering Institute, University of Auckland, Auckland, New Zealand; ^2^ Department of Engineering Science and Biomedical Engineering, University of Auckland, Auckland, New Zealand

**Keywords:** Ca^2+^ sensitivity, myofilament, calcium, crossbridge, experimental techniques, biophysical modelling

## Abstract

Myofilament calcium (Ca^2+^) sensitivity is one of several mechanisms by which force production of cardiac muscle is modulated to meet the ever-changing demands placed on the heart. Compromised Ca^2+^ sensitivity is associated with pathologies, which makes it a parameter of interest for researchers. Ca^2+^ Sensitivity is the ratio of the association and dissociation rates between troponin C (TnC) and Ca^2+^. As it is not currently possible to measure these rates in tissue preparations directly, methods have been developed to infer myofilament sensitivity, typically using some combination of force and Ca^2+^ measurements. The current gold-standard approach constructs a steady-state force-Ca^2+^ relation by exposing permeabilised muscle samples to a range of Ca^2+^ concentrations and uses the half-maximal concentration as a proxy for sensitivity. While a valuable method for steady-state investigations, the permeabilisation process makes the method unsuitable when examining dynamic, i.e., twitch-to-twitch, changes in myofilament sensitivity. The ability of the heart to transiently adapt to changes in load is an important consideration when evaluating the impact of disease states. Alternative methods have been proffered, including force-Ca^2+^ phase loops, potassium contracture, hybrid experimental-modelling and conformation-based fluorophore approaches. This review provides an overview of the mechanisms underlying myofilament Ca^2+^ sensitivity, summarises existing methods, and explores, with modelling, whether any of them are suited to investigating dynamic changes in sensitivity. We conclude that a method that equips researchers to investigate the transient change of myofilament Ca^2+^ sensitivity is still needed. We propose that such a method will involve simultaneous measurements of cytosolic Ca^2+^ and TnC activation in actively twitching muscle and a biophysical model to interpret these data.

## 1 Introduction

Calcium (Ca^2+^) sensitivity is a critical mechanism by which force production of cardiac muscle is modulated to meet the ever-changing demands placed on the heart. As the ventricles of the heart form an electrical syncytium (i.e., the entire ventricle contracts with each beat), the work performed by cardiac muscle cannot be regulated through mechanisms like recruitment, as is the case in skeletal muscle. Instead, changes in frequency, stroke volume, and contractility represent the cornerstones of cardiac output regulation. Ca^2+^ sensitivity falls under the umbrella of mechanisms that modulate contractility.

Due to the functional importance of Ca^2+^ sensitivity and its disruption in pathologies, particularly in diabetic cardiomyopathy (Bielefeld et al., 1983), dilated cardiomyopathy (Wolff et al., 1996), and hypertrophic cardiomyopathy (Frey et al., 2012), the scientific community has developed several methods for assessing sensitivity. Generally, those methods require simultaneous measurements of cytosolic Ca^2+^ concentration (hereon [Ca^2+^]_i_) and one of length or force. To our knowledge, the earliest force-Ca^2+^ curve of cardiac muscle was obtained from skinned muscles by the seminal study of Fabiato and Fabiato (1975), following a demonstration of the force-Ca^2+^ curve obtained from glycerated ventricular rings (Bozler, 1968) and curvature relations between ATPase activity and Ca^2+^ in cardiac myosin by Katz and colleagues in the 1960s (Katz and Repke, 1966; Katz, 1967). Since then, methods have been developed to accommodate intact muscle preparations from animals of varying sizes (e.g., mouse (McDonald et al., 1995; Rajan et al., 2007) to human (Van der Velden et al., 2000; Gomes et al., 2005)), which are more physiologically appropriate than their permeabilised counterparts.

This review focuses on *ex-vivo* methods used by the scientific community to assess cardiac myofilament Ca^2+^ sensitivity. We first summarise the knowledge and concepts that underpin myofilament calcium sensitivity before examining the various measurement methods that have been used to probe this characteristic in *ex-vivo* heart tissue. These include identification techniques using chemical and electrical stimuli, mathematical model-based analyses, and recently developed fluorescent probes. We outline necessary considerations for each of the methods and speculate on the future direction of *ex-vivo* methods for assessing myofilament Ca^2+^ sensitivity.

## 2 Subcellular basis of cardiac myofilament Ca^2+^ sensitivity

An in-depth description of the modulatory mechanisms of Ca^2+^ sensitivity has been provided in existing reviews (Allen and Kentish, 1985; Chung et al., 2016). We, thus, provide a brief overview of the key proteins involved in the Ca^2+^-based regulation of cardiac myofilament interaction to define the nomenclature used throughout the review.

We start with the structures involved in force production and Ca^2+^-based regulation. “Myofilaments” refers to strand-like protein complexes whose interaction results in force production. In a reductive sense, there are two types of myofilaments: the thin filament, which consists of actin, the troponin complex, and tropomyosin, and the thick filament, which consists of mainly myosin molecules (De Tombe, 2003). Another important protein in force regulation is myosin-binding protein C, which regulates the structural arrangement of the lattice (Knöll, 2012). In cardiac muscle, force production occurs from crossbridge cycling–a process whereby myosin heads of the thick filament cyclically interact with actin molecules of the thin filament. At low [Ca^2+^]_i_, troponin and tropomyosin prevent actin and myosin from interacting (Tobacman, 2021). The troponin complex comprises three subunits: troponin C (TnC), troponin I (TnI), and troponin T (TnT). The arrival of an action potential induces [Ca^2+^]_i_ to increase due to extracellular and intracellular Ca^2+^ fluxes. Ca^2+^ binding to TnC causes a conformational change in the thin filament, removing the steric hindrance and enabling strong myosin-actin binding.

## 3 Sensitivity versus responsiveness

Augmented Ca^2+^ sensitivity has, at times, been misrepresented as greater force production for a given [Ca^2+^]_i_ (Bers, 2002). The authors of this review would like to demarcate myofilament-centric Ca^2+^ sensitivity from cross-bridge-centric responsiveness, as was the historical definition (Marban and Kusuoka, 1987). TnC-Ca^2+^ binding affinity, or the ratio of Ca^2+^ attachment and detachment rates, is Ca^2+^ sensitivity, which can be modulated through post-translational modifications like the phosphorylation of TnI (Salhi et al., 2016; Bowman et al., 2019). In contrast, cross-bridge-centric mechanisms, such as the number of available cross-bridges and unitary force (force generated per cross-bridge (De Tombe and Ter Keurs, 2016)), are mechanisms of “responsiveness”. Cooperativity represents a grey area of cross-bridge- and myofilament-centric mechanisms, where bound cross-bridges increase the Ca^2+^ binding affinity of proximal TnC sub-units. Hopefully, the issue with the pervasive definition of Ca^2+^ sensitivity is now apparent; it conflates responsiveness, cooperativity, and sensitivity.

**FIGURE 1:**
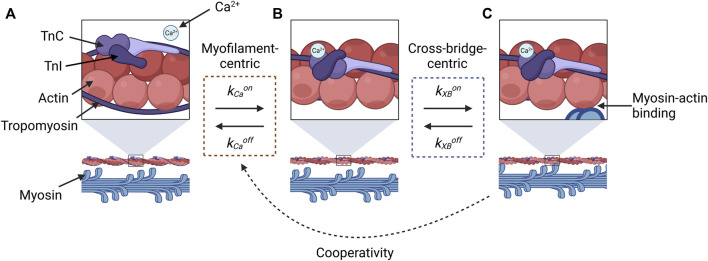
Illustration of reaction states for cross-bridge formation that are relevant for evaluating the constituent components of force-Ca^2+^ dynamics. State **(A)** represents the absence of Ca^2+^, and the myosin-binding sites of actin molecules (top panel) are sterically inhibited by tropomyosin and the troponin complex, so myosin-actin binding does not occur (lower panel). Once Ca^2+^ binds to TnC, a conformational change in tropomyosin and troponin removes the steric hindrance **(B)**, which makes actin-myosin binding possible **(C)**. The transition between **(A)** and **(B)** is described by the kinetic rates for the association (*k*_Ca_^on^) and dissociation (*k*_Ca_^off^) of Ca^2+^ to and from TnC, respectively (orange dashed box). The transition between States **(B)** and **(C)** is described by the kinetic rates associated with the attachment (*k*_XB_^on^) and detachment (*k*_XB_^off^) of cross-bridges (blue dashed box). Force-dependent cooperativity is indicated by the dotted black arrow, where the force by crossbridges modulates Ca^2+^-TnC binding affinity. This figure was created with BioRender.com.

This conflation has likely arisen partly because of the challenges associated with inferring sensitivity from force and Ca^2+^ measurements, as most of the methods discussed in this review have done. Let us consider the reaction states of a myofilament in a three-state representation (Figure 1): **A**, no Ca^2+^ is bound to TnC, so myosin and actin are unable to bind to form a cross-bridge; **B**, Ca^2+^ is bound to TnC and the tropomyosin-troponin complex has undergone a conformational change, but myosin and actin have not yet bound; and **C**, myosin and actin are strongly bound. When measuring the effect of Ca^2+^ on force, the only state we can measure is State **C**, which manifests, indirectly and macroscopically, as force production. Hence, we cannot differentiate between the changes in sensitivity that are driven by myofilament-centric processes (between States **A** and **B**) and those driven by cross-bridge-centric processes (between States **B** and **C**).

In addition, there is a temporal delay between any change in [Ca^2+^]_i_ and the number of strongly bound cross bridges, as the transition from State **A** to State **B** and State **B** to State **C** is not instantaneous. To infer the ratio of *k*_*Ca*_^*on*^ and *k*_*Ca*_^*off*^ (sensitivity) using force information alone requires both reactions to be in equilibrium. If not, the number of bound cross-bridges will be rate-limited by time dynamics and will not reflect the underlying binding affinities. The notion of equilibrium and steady state is essential for the sensitivity assessment methods discussed below.

## 4 Steady-state force-Ca^2+^ relation

**FIGURE 2:**
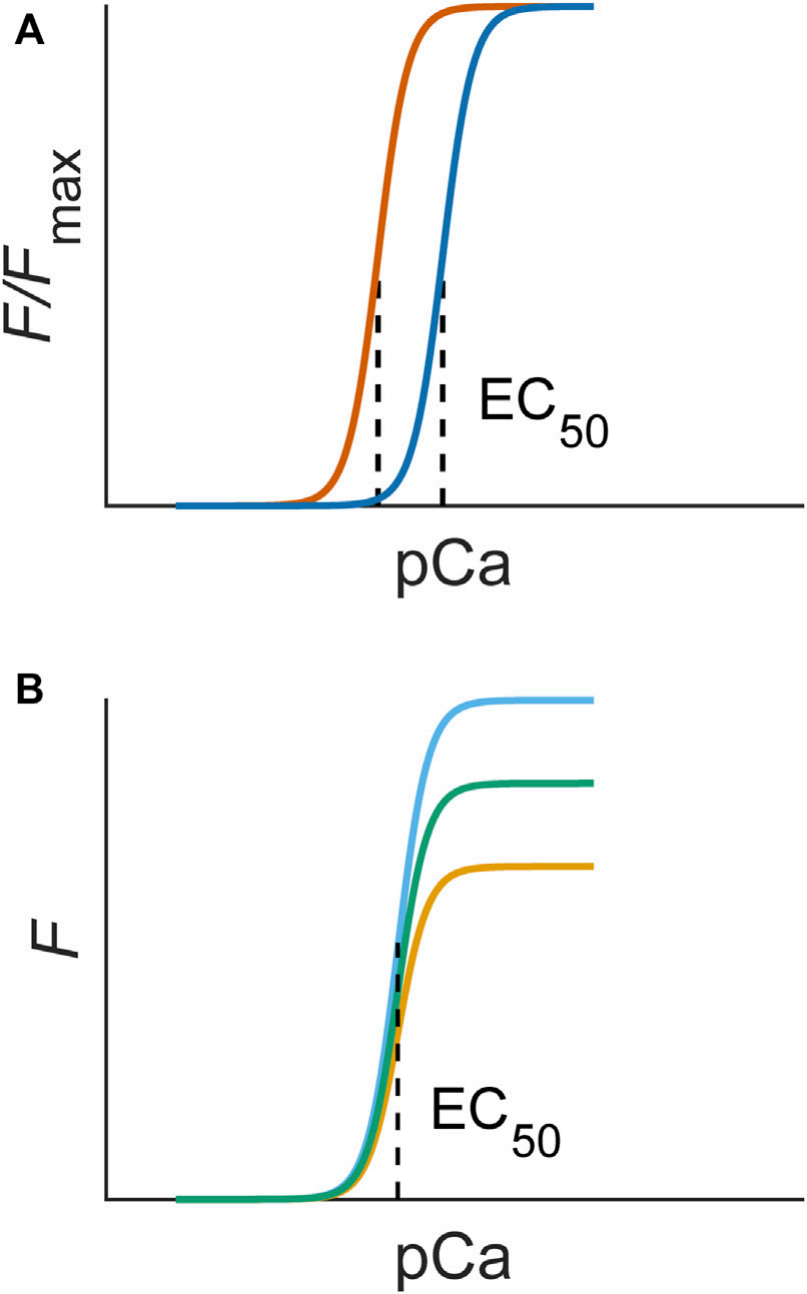
**(A)** An increase in myofilament sensitivity is associated with a left shift of the force-Ca^2+^ curve (blue to orange), quantifiable as EC_50_. **(B)** A change in responsiveness alone would not change sensitivity and, hence, would not change the EC_50_ of steady-state force-Ca^2+^ curves. The Hill equation (Eq. 1) used to fit experimental data contains these parameters: *F*_max_ is the maximum force, *n* is the Hill coefficient, which indicates the steepness, or cooperativity, of the relation, and EC_50_ is the concentration of Ca^2+^ associated with half-maximal force production (dotted line).

The most used method for inferring myofilament Ca^2+^ sensitivity utilises the steady-state force-Ca^2+^ relation. This relation is constructed by measuring the steady-state force production of a muscle sample over a range of prescribed or measured Ca^2+^ concentrations, typically expressed as pCa (Figure 2). Such an experiment can be conducted under a range of temperatures and sarcomere lengths (Kentish et al., 1983; Harrison and Bers, 1989). These data are typically fitted to a Hill equation, where EC_50_ (also *k*_*d*_) is defined as the Ca^2+^ concentration associated with half-maximal force production and *n* is the Hill coefficient, a measure of the steepness, or cooperativity, of the relation (e.g., Equation 1). The force produced is normalised to the peak value (*F*_max_) obtained under the highest concentration of Ca^2+^. A lower EC_50_ represents an increased Ca^2+^ sensitivity, as a lesser Ca^2+^ concentration is required for the sample to reach half-maximal force production (Figure 2A). This technique has been performed using permeabilised (Fabiato and Fabiato, 1975; Wolff et al., 1996) and intact (Gao et al., 1994; Varian et al., 2006) muscle preparations.

$$F=\frac{F_{\max}\times {\left({\left[Ca^{2+}\right]}_{i}\right)}^{n}}{{\left({\left[Ca^{2+}\right]}_{i}\right)}^{n}+{\left(EC_{50}\right)}^{n}}$$ (1)

As mentioned in the introduction, Fabiato and Fabiato (1975) performed the first example of this method using skinned (or permeabilised) samples. The skinning process removes all sarcolemmal structures by chemical (e.g., triton or saponin) or mechanical means, exposing the contractile myofilament proteins to the bathing solution. In this way, skinning can be thought of as a means of homogenising the extracellular and intracellular compartments. Permeabilisation is known to increase myofilament lattice spacing (Matsubara, 1980), which is thought to decrease the probability of cross-bridge formation (McDonald and Moss, 1995). This concern is mitigated by the use of a high-molecular-weight polymer, such as dextran (2%–3%), to compress the myofilament lattice back to near physiological levels (Irving et al., 2000). After skinning, permeabilised muscles are no longer capable of producing a force twitch. Instead, these samples produce a sustained contracture force in response to the presented Ca^2+^ concentration in the bathing solution. A secondary benefit of leveraging permeabilised samples is the ability to use frozen samples with minimal impact on the apparent myofilament Ca^2+^ sensitivity (Milburn et al., 2022), which enables the use of human samples from existing tissue banks. Steady-state length-Ca^2+^ relations have also been used to assess myofilament Ca^2+^ sensitivity in skinned cardiac myocytes (Siri et al., 1991; Lim et al., 2001), though this is uncommon. Achieving the low Ca^2+^ concentration needed to assess the entire relation (∼1 nM) necessitates the use of a chelating agent, such as EGTA or BAPTA (Bers et al., 2010). Free [Ca^2+^] is estimated using specialist software, which account for variables like pH, ionic strength, and temperature (Brooks and Storey, 1992; Dweck et al., 2005). For each [Ca^2+^], the permeabilised sample is left in the Ca^2+^ solution until a steady-state force (or length) is reached, which is to say that the relation is the direct result of binding kinetics instead of a conflation of rate-limited reactions.

In an intact muscle, where the sarcolemma is maintained, intracellular and extracellular Ca^2+^ concentrations are not equivalent. As a result, [Ca^2+^]_i_ concentration must be measured using a fluorescent indicator (e.g., Fura-2 (Grynkiewicz et al., 1985)). The bathing solution used for these studies also commonly contains caffeine or ryanodine, which enables tetanic contractions by limiting the sequestering of Ca^2+^ from the cytosolic space (Allen and Westerblad, 1995). Ryanodine has been shown to have no effect on myofilament Ca^2+^ sensitivity (Fabiato, 1985), but caffeine increases myofilament Ca^2+^ sensitivity (Wendt and Stephenson, 1983). Hence, when using tetani-based contracture to assess the steady-state force-Ca^2+^ relation, ryanodine should be used to inhibit the sequestering of Ca^2+^ within the SR. As in skinned experiments, force and intracellular Ca^2+^ are measured simultaneously during tetani over a range of extracellular Ca^2+^ concentrations. The extent of shortening of intact cells undergoing a tetanic contraction has also been used to assess sensitivity (Hongo et al., 1998).

The steady-state force-Ca^2+^ relations and, therefore, myofilament sensitivity, measured using intact and skinned samples are not equivalent, with intact samples appearing to have a greater Ca^2+^ sensitivity (Gao et al., 1994; Varian et al., 2006). However, skinning does not appear to negatively affect the maximum force production of cardiac muscle (Choi et al., 2020). The discrepancy in Ca^2+^ sensitivity has instead been speculatively attributed to a loss of post-translational modifications caused by the permeabilisation process (Chung et al., 2016). Hence, intact assessment of myofilament Ca^2+^ sensitivity appears preferable for steady-state sensitivity measurements.

## 5 Potassium contracture

**FIGURE 3:**
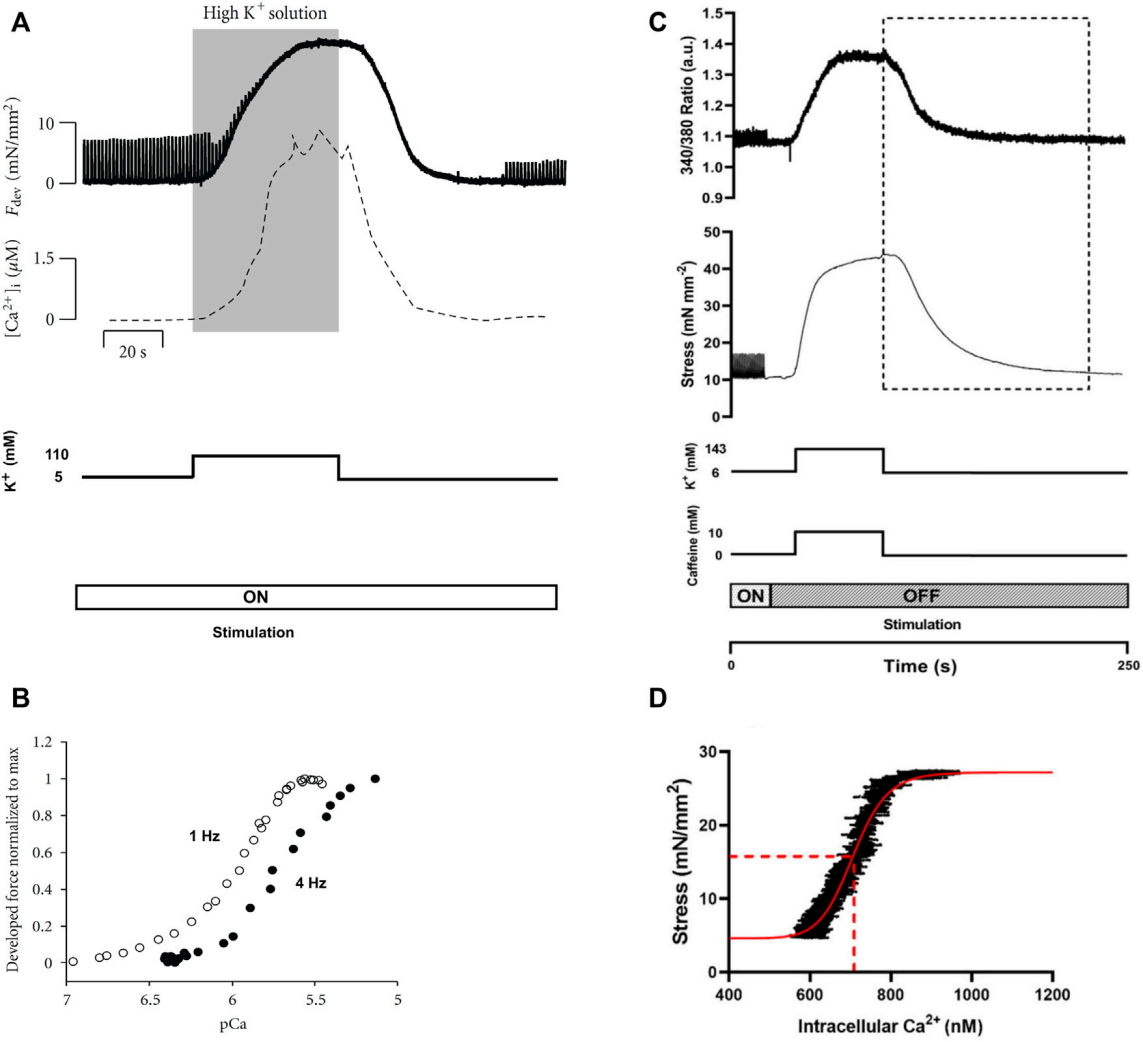
Potassium contracture techniques. Panels **(A)** and **(B)** are replicated from Varian et al. (2012). Panels **(C)** and **(D)** are reproduced from Jones et al. (2023). **(A)** The contracture protocol of Varian et al. exposes muscle to a high K^+^ superfusate solution (shaded region), with the electrical stimulation (at 1 Hz) remains on (Varian et al., 2012). Force twitches diminish upon contracture and re-develop upon washout of the high K^+^ superfusate. **(B)** Force-[Ca^2+^]_i_ data were obtained from the rising (upstroke) phase of the contracture (shaded region in panel **(A)**. The 4 Hz data were positioned left to that of the 1 Hz, which was interpreted by Varian et al. as reduced myofilament Ca^2+^ sensitivity (i.e., frequency-dependent myofilament desensitization). **(C)** The high-K^+^ contracture protocol of the Ward lab (Jones et al., 2023) includes high caffeine with the electrical stimulation halted. **(D)** Force-[Ca^2+^]_i_ data were taken from the downstroke phase of the contracture (indicated by the dotted rectangle in panel **(C)** for curve fitting (red line) for the estimation of EC_50_ (dotted red line).

The potassium (K^+^) contracture technique was developed by Varian et al. (2006) to measure steady-state myofilament Ca^2+^ sensitivity using intact muscle preparations (Figure 3A). The protocol involves switching the superfusate buffer to a modified composition with elevated KCl concentration (100 mM–150 mM). The high level of extracellular K^+^ evokes muscle contracture by depolarization of the cell membrane (Niedergerke, 1956); the force of contracture is proportional to the ratio of [Ca^2+^] to the square of [Na^+^] in the superfusate (Lüttgau and Niedergerke, 1958). Thus, the modified superfusate contains an increased concentration of CaCl_2_ (around 5 mM) and a reduced concentration of NaCl (around 40 mM). The contracture ceases upon return to normal superfusate solution. The contracture takes about 5 min to complete.

Varian et al. (2006) simultaneously measured [Ca^2+^]_i_ and showed comparable up and down strokes of Ca^2+^ to that of the contracture force. As quoted from the authors, both [Ca^2+^]_i_ and force ‘*rise and fall at nearly the same rate*’ (Figure 3A). However, only the rising phase up to the maximum force of contracture (i.e., upstroke) has been used to estimate myofilament Ca^2+^ sensitivity from the resulting sigmoidal curve (Figure 3B). The sigmoidal force-Ca^2+^ curves from the upstroke and downstroke have not been compared, so it remains to be seen if they are equivalent.

Pulsatile electrical stimulation remains throughout the contracture, such that the muscle can be paced at various stimulation frequencies. This stimulation protocol allowed Varian et al. to conclude that myofilament Ca^2+^ sensitivity decreases with increasing frequency (i.e., a rightward shift of the force-[Ca^2+^]_i_ curve; Figure 3B) (Varian and Janssen, 2007). Such a modulatory role of frequency-dependent myofilament desensitization was found to be mediated through a kinase-specific pathway involving phosphorylation of myofilament proteins (Varian et al., 2012), and to be impaired in ventricular hypertrophy (Varian et al., 2009).

Although potassium contracture was developed almost two decades ago, it has not gained widespread popularity within the scientific community. Only one other research group has since adopted the technique (Kaur et al., 2016; Jones et al., 2023), albeit with three adjustments (Figure 3C). First, the elevated KCl in the superfusate is supplemented with caffeine (10 mM) to increase [Ca^2+^]_i_ through induced release of Ca^2+^ from the SR. Note that caffeine changes myofilament sensitivity (Wendt and Stephenson, 1983). Second, stimulation is halted throughout the contracture. Third, the downstroke of the contracture is used instead for constructing the force-Ca^2+^ curve (Figure 3D).

The K^+^ contracture protocol has been performed with variations that are specific to the study aim. Varian et al. aimed to assess the frequency-dependence of myofilament Ca^2+^ sensitivity and, hence, required stimulation and the upstroke of the K^+^ contracture was used. In contrast, Ward et al. focused on Ca^2+^ handling; hence, caffeine was added, and the downstroke of the K^+^ contracture was used. A consideration for future use of the K^+^ contracture technique will include whether the upstroke and downstroke produce the same force-Ca^2+^ curve, given the knowledge that the upstroke positively correlates with the prior stimulation frequency (Niedergerke, 1956; Gibbons and Fozzard, 1971).

It is not immediately clear as to why this method has not gained widespread popularity. Monasky et al. (2010), from the Janssen lab that pioneered this technique, commented that ‘*the potassium contracture based assessment of calcium sensitivity is more technically challenging* [than permeabilised steady-state analysis] *and has a lower experimental success rate’.* We speculate that these factors, combined with the relative ease with which force-Ca^2+^ phase-plane loop analysis can be performed (often with an insufficient appreciation of their limitations; see “Twitch-Based Methods”), have contributed to the poor uptake of potassium contracture as a method for evaluating myofilament Ca^2+^ sensitivity.

## 6 Twitch-based methods

With the advent of fluorescent indicators, initially aequorin (Shimomura, 1995), then Fura−2 (Grynkiewicz et al., 1985), investigators sought to leverage the new capability of simultaneous force (or length) and intracellular Ca^2+^ measurements to assess Ca^2+^-based contractile regulation. These techniques use force and Ca^2+^ measurements recorded in twitching intact muscle tissue (e.g., papillary muscles or trabeculae) or single-cell preparations. Each technique described in this section can be applied to a single twitch.

### 6.1 Using a single data point

**FIGURE 4:**
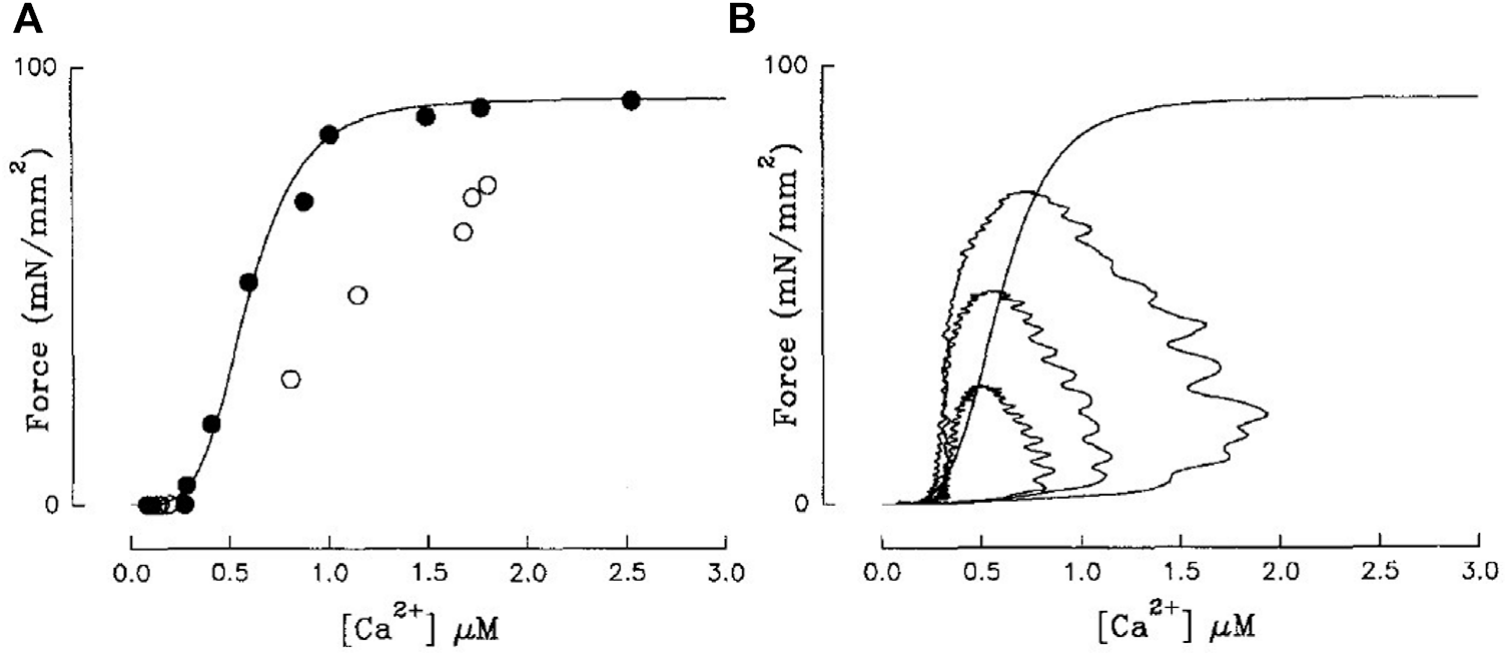
**(A)** Steady-state force-Ca^2+^ (black) and peak-force-Ca^2+^ relations (white) are not equivalent. **(B)** Isometric force-Ca^2+^ phase-plane loops at three different [Ca^2+^]_o_ are also not equivalent to the steady-state relation (sigmoid). Steady-state force-Ca^2+^ relation was measured using ryanodine-induced tetani. Reproduced from Backx et al. (1995).

For a time, twitch-based methods used relations derived from single time points of Ca^2+^ and contractile (i.e., force or length) traces to infer sensitivity. Early work by Blinks and colleagues hypothesised that the peak of a Ca^2+^ transient and associated twitch force amplitude, while dissociated in time, corresponded closely to the steady-state force-Ca^2+^ relation (Endoh and Blinks, 1988; Blinks, 1993). Backx et al. (1995) subsequently disproved this hypothesis by plotting the peak-force-Ca^2+^ relation on the same axis as tetanic (ryanodine) force-Ca^2+^ and illustrating that they were quite different (Figure 4A). In fact, empirical findings indicate that any shift in the peak Ca^2+^-force relation results from a modulation in the time course of the Ca^2+^ and force twitch rather than a change of myofilament Ca^2+^ sensitivity (Hajjar et al., 1992).

As with steady-state relations, researchers have also considered Ca^2+^-length relations with single data point approaches. Siri et al. (1991) plotted sarcomere length measured in isolated cells not loaded with Fura-2 against [Ca^2+^]_i_ measured in other cells from the same heart paced at the same stimulation frequency. They plotted two collections of data points per group, one between diastolic sarcomere length and Ca^2+^ and the other between systolic sarcomere length and peak Ca^2+^. The range of [Ca^2+^]_i_ over which relations were plotted was achieved by leveraging the frequency dependency of Ca^2+^ transient amplitude. It should be noted that TnI phosphorylation, and therefore myofilament Ca^2+^ sensitivity, has since been shown to be frequency dependent (Varian and Janssen, 2007). When comparing controls and an animal model of congestive heart failure (aortic-banded guinea pigs), they observed that the Ca^2+^-sarcomere length relations for each group overlayed, indicating no sensitivity shift (Siri et al., 1991). Besides the challenge of collecting sarcomere length and [Ca^2+^]_i_ information from different cells, a temporal offset between peak Ca^2+^ and systolic length is also present, leading to the same issue as in the force-equivalent method.

### 6.2 Phase-plane loops

Phase-plane loops are generated by parametrically plotting either force or length against [Ca^2+^]_i_ (Figure 4B). A component of the phase-plane loop, typically the relaxation phase, is used to indicate myofilament Ca^2+^ sensitivity. The linear gradient (Ward et al., 2010), EC_50_ (Gao et al., 1998; Jones et al., 2023), and qualitative assessment of the trajectory of the relaxation phase (Kentish and Wrzosek, 1998; Zhao et al., 2016) have all been considered as sensitivity indicators. The relaxation phase is the predominant focus for this technique because this is the part of the twitch with the slowest rate of change of Ca^2+^, suggesting it has the greatest chance of achieving force-Ca^2+^ equilibrium. However, the non-linear nature of the relaxation phase means the linear gradient is sensitive to the position along the relaxation phase where it is measured. Further, EC_50_ conflates Ca^2+^ handling with myofilament and crossbridge effects (i.e., a large change in diastolic Ca^2+^ or Ca^2+^ amplitude would be interpreted as a change of sensitivity regardless of TnC-Ca^2+^ binding affinity).

In fact, cynicism has plagued the acceptability of these methods since their inception, with numerous studies speculating that force and Ca^2+^ are not in equilibrium throughout the loop (Yue et al., 1986; Yue, 1987; Endoh and Blinks, 1988; McIvor et al., 1988). Subsequent studies affirmed these suspicions by demonstrating that mechanical loading decouples the force and Ca^2+^ signals in the time domain (Peterson et al., 1988; Brutseart and Sys, 1989; Spurgeon et al., 1992). Spurgeon et al. (1992) suggested that, under isometric conditions, equilibrium between force and Ca^2+^ is reached only at the end of diastole. As expected, force-Ca^2+^ phase-plane loops sit on a different relation from that of steady-state (Figure 4B; (Backx et al., 1995)). Substantially slowing the sequestering of Ca^2+^ and the downstroke of the Ca^2+^ transient, using cyclopiazonic acid (SERCA inhibitor) brought the two relations into alignment (Backx et al., 1995), reinforcing the lack of equilibrium under physiological conditions. Despite these criticisms, force-Ca^2+^ phase-plane loops are still used to infer myofilament sensitivity (Kentish and Wrzosek, 1998; Han et al., 2010; Ward, 2014; Jones et al., 2023), given the conjecture that this approach can predict sensitivity under conditions known to modify sensitivity (Ward, 2014; Jones et al., 2023). This technique is better used to qualitatively assess disruptions to the time course of Ca^2+^ cycling (Pérez et al., 1999).

**FIGURE 5:**
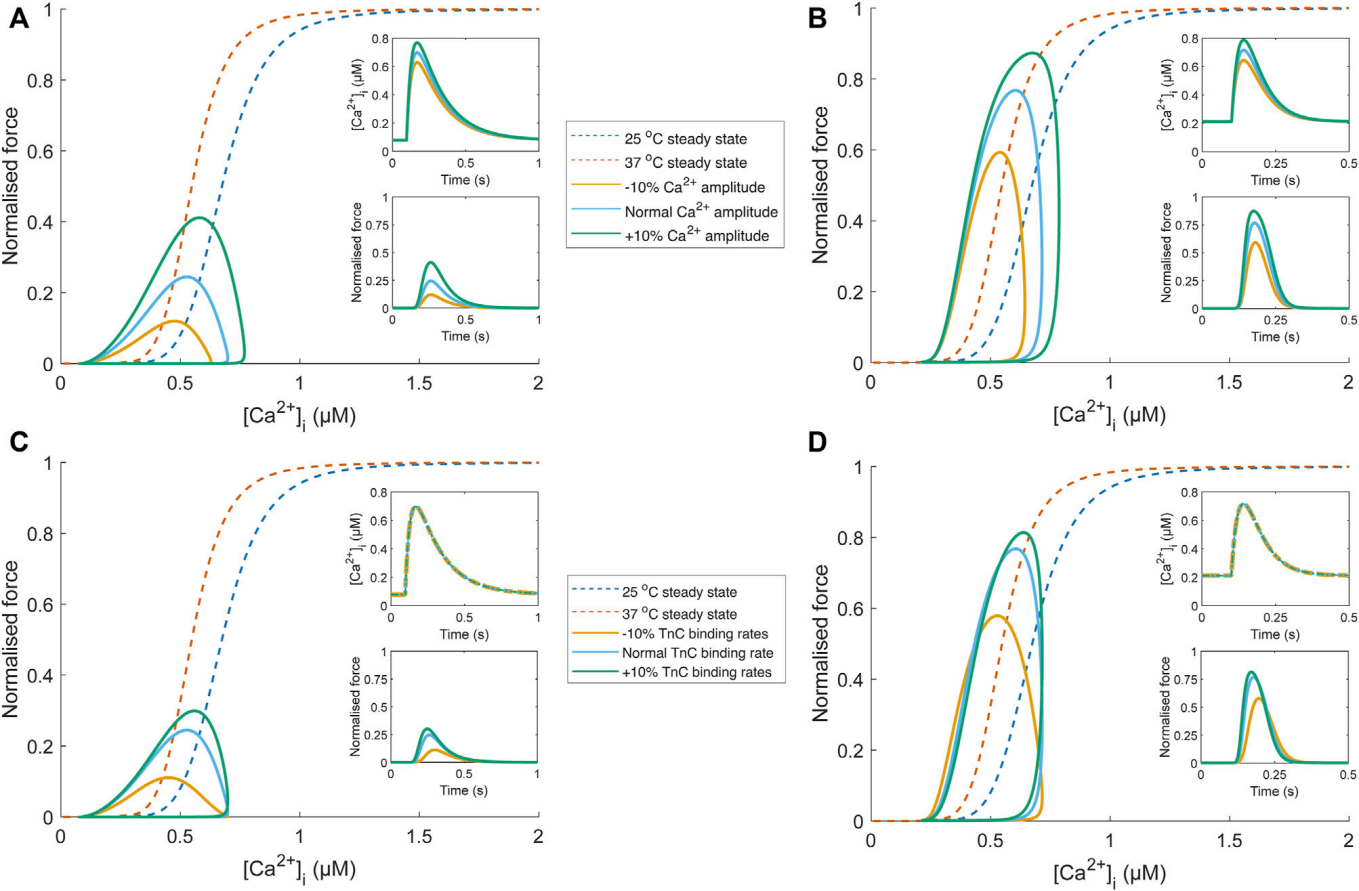
Comparison of phase-plane loop morphology under conditions where Ca^2+^ sensitivity is unchanged using a biophysical model (Rice et al., 2008). Temperature-dependent Ca^2+^ transients (25°C and 37°C) were obtained from Chung and Campbell (2013), scaled to align with the force-Ca^2+^ relation of the biophysical model (blue (25°C) and orange (37°C) dashed lines), and fit with a Ca^2+^ transient function, defined by two decaying exponentials with parameters τ_1_, τ_2_, Ca_dias_, Ca_amp_, and *t*_*start*_ (see Appendix of Rice et al. (2008)). The parameter set for the “normal” Ca^2+^ transient at each temperature was τ_1_ = 34.9 ms, τ_2_ = 183.3 ms, Ca_dias_ = 0.078 µM, Ca_amp_ = 0.70 µM, and *t*_*start*_ = 96.4 ms (25°C Ca^2+^ transient); τ_1_ = 54.4 ms, τ_2_ = 31.8 ms, Ca_dias_ = 0.21 µM, Ca_amp_ = 0.72 µM, and *t*_*start*_ = 100 ms (37°C Ca^2+^ transient). The influence of Ca^2+^ handling on phase-plane loop morphology is illustrated in panels **(A)** and **(B)**, where the amplitude of the “normal” temperature-dependent Ca^2+^ transient (blue) was increased (green) and decreased (yellow) by 10% by changing the Ca_amp_ parameter. These Ca^2+^ transients were then provided as an input to the biophysical model (upper insets; **(A)** and **(B)**. The resultant force output of the model (lower insets; **(A)** and **(B)** was plotted against the Ca^2+^ transient, and despite no change to myofilament sensitivity, modulation of Ca^2+^ transient amplitude results in different phase-plane loop morphologies (EC_50_ and relaxation phase gradient) at 25°C **(A)** and 37°C **(B)**. Concomitant changes (i.e., all changed by the same factor) of TnC-Ca^2+^ binding rates in the biophysical model (*k*_*on*_, *k*_*offHT*_, and *k*_*offLT*_) also influence the phase-plane loop morphology at 25°C **(C)** and 37°C **(D)** when the same temperature-dependent Ca^2+^ transient is provided as input (upper insets; **C** and **D**).

To emphasise the fallibility of force-Ca^2+^ phase-plane loops as an indicator of myofilament Ca^2+^ sensitivity, we have included *in silico* analyses focusing on conditions that modify phase-plane loop morphology in the absence of a change in myofilament Ca^2+^ sensitivity. Using a detailed mathematical model of Ca^2+^-force dynamics (Rice et al., 2008), we show that phase-loop morphology is influenced when only Ca^2+^ handling changes (Figures 5A,B). As temperature increases, the relaxation phase gradient approaches that of the steady-state relation, though Ca^2+^ amplitude remains influential. EC_50_ is also an inappropriate indicator of myofilament sensitivity because of the undue influence of Ca^2+^ transient amplitude. Further, an equal change to TnC-Ca^2+^ binding rates (i.e.*,* modifying association and dissociation rates change by the same factor) is not associated with a change of myofilament Ca^2+^ sensitivity but manifests as a change of phase-plane loop morphology (Figures 5C,D). While changes in Ca^2+^ sensitivity would also lead to a change in phase-plane loop morphology, it is uncertain which aspect of the loop should be considered as an indicator due to the conflation of many other factors. Despite their convenience, phase-plane loops are unsuitable for quantifying myofilament Ca^2+^ sensitivity.

In light of the foibles of force-Ca^2+^ phase-plane loops, Spurgeon et al. (1992) hypothesised that single cells undergoing unloaded shortening would be under equilibrium throughout the entire re-lengthening phase. This technique uses single-cell preparations, and unloaded shortening is plotted against Ca^2+^ (Graham et al., 2005; Zhao et al., 2016). As with the force-Ca^2+^ phase plane loops, EC_50_ (Zhao et al., 2016), linear gradient (of the final linear region) (Graham et al., 2005), and qualitative assessment of the trajectory (Spurgeon et al., 1992) have been used to infer myofilament sensitivity. Zhao et al. (2016) removed the diastolic length and Ca^2+^ effects by translating the bottom-left data point of their signals to the origin (i.e., minimum sarcomere length and Fura-2 fluorescence set to zero). However, Ca^2+^ transient amplitude would still have too large an influence on EC_50_ for this to be a consistently useful indicator. Of course, the length dependency of sensitivity (De Tombe et al., 2010) presents an additional challenge.

## 7 Model-based assessment

A modelling-based approach to infer myofilament Ca^2+^ sensitivity would involve fitting experimental data with a biophysical cardiac model and calculating the TnC-Ca^2+^ binding rates. Biophysical cardiac models represent Ca^2+^ and myofilament dynamics as biochemical reactions with mass action kinetics, where the kinetic rate of a reaction is proportional to the concentration of the reactants. Mass action kinetics are used to model the binding interactions between Ca^2+^, TnC, and myosin and the transition of tropomyosin and the troponin complex between different configurations or states. These states evolve dynamically and are described by a set of ordinary differential equations, which are solved to predict the behaviour of the system over time. Each state represents a fractional occupancy of TnC in a given configuration; the states sum to one because TnC is conserved in the system. The transition between states is governed by two kinetic rates: one in the forward direction and one in the reverse direction. The ratio between the forward and reverse rates determines the steady-state ratio between two states.

The complexity of a model depends on the number of states and the formulation of the kinetic rates. The ability to uniquely identify model parameters from experimental data depends on the model complexity and the information content of the data (Shames et al., 1996). For an inotropic response where the input Ca^2+^ transient is unchanged, the increase in force/pressure can be due to either an increase in Ca^2+^ affinity for TnC (sensitivity) or an increase in the rate of cross-bridge formation (responsiveness). A Ca^2+^-myofilament model that is considered identifiable would be able to distinguish between these two effects. For a given data set, the higher the complexity, the greater the number of parameters, and the less identifiable the model becomes.

**FIGURE 6:**
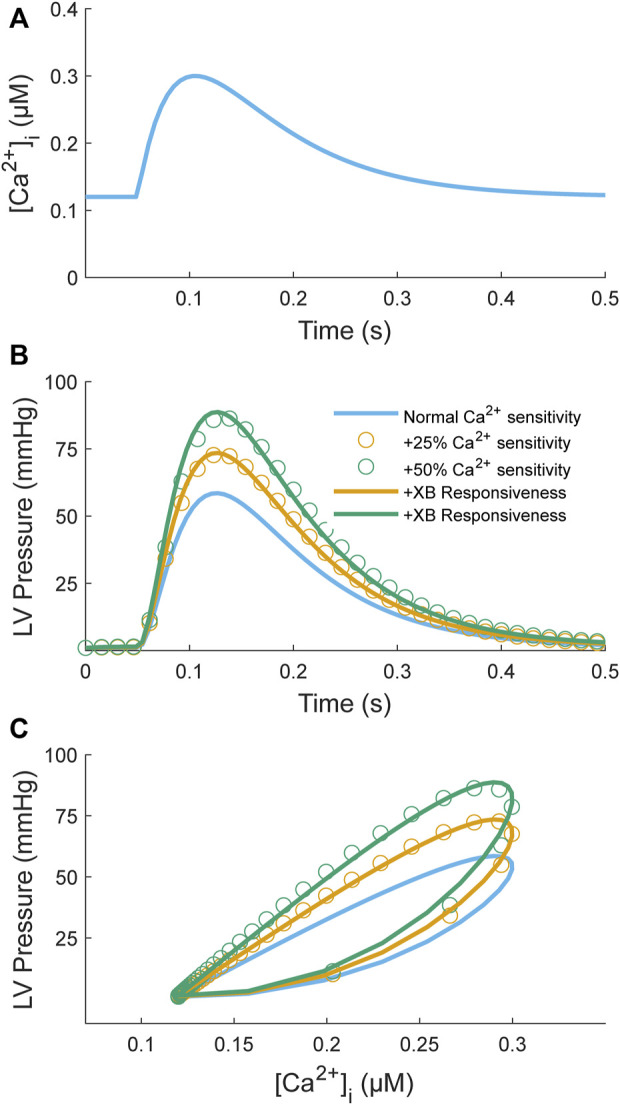
Model simulation of an increase in Ca^2+^ myofilament sensitivity to test identifiability. The myofilament model (Op Den Buijs et al., 2008) takes a Ca^2+^ transient **(A)** as input and produces an LV pressure transient **(B)** as output; a parametric plot of these two variables produces a phase-loop **(C)**. The Ca^2+^ transient function is defined by two decaying exponentials with parameters τ_1_ = 80 ms, τ_2_ = 40 ms, Ca_dias_ = 0.12 µM, Ca_amp_ = 0.3 µM, and *t*_*start*_ = 50 ms (see Appendix of Rice et al. (2008)). The myofilament model was used to simulate two sets of positive inotropic responses corresponding to an increase in the Ca^2+^ binding affinity by 25% (orange open circles) or 50% (green open circles) relative to control (blue line). The model parameters for the control model were taken from Table 2 of Op Den Buijs et al. (2008): *k*_1_ = 38 s^−1^, *k*_3_ = 361 s^−1^, α = 0.29 nM^−2^s^−1^, β = 658, *k*_d_ = 212 s^−1^, and Ca_t_ = 117 nM. For generation of the simulation data, all parameters were kept the same except for *k*_1_, which was increased by either 25% or 50%. The model was then fitted individually to these two data sets by constraining the Ca^2+^ parameters (*k*_1_, *k*_3_, Ca_t_) to be unchanged from control and allowing only the cross-bridge parameters to be modified (α, β, *k*_d_). The resulting fitted curves are illustrated as orange or green lines, respectively. The objective was to assess whether the inotropic response induced by increasing Ca^2+^ binding affinity can be replicated by only a change in cross-bridge kinetics. For both cases, parameter sets were found where the NRMSEs were below 0.4%. The parameter values were α = 0.82 nM^−2^s^−1^, β = 1,505, *k*_d_ = 390 s^−1^ (orange line) and α = 1.45 nM^−2^s^−1^, β = 3,280, *k*_d_ = 703 s^−1^ (green line) for a 25% and 50% increase in *k*_1_, respectively.

One study has proposed a model-based framework explicitly for assessing myofilament Ca^2+^ sensitivity using a simple two-state, seven-parameter model (Op Den Buijs et al., 2008). This model is highly simplified compared to others in the literature (Rice et al., 2008) in an effort to improve identifiability. Here, we want to determine whether such a model is identifiable given simulated data sets consisting of Ca^2+^ transients as inputs and force/pressure responses as outputs. We first used an input Ca^2+^ transient (Figure 6A) to simulate a control pressure response (Figure 6B; blue line). Model parameters are from Table 2 of Op Den Buijs et al. (2008). We then simulated two sets of inotropic responses by increasing the parameter governing the Ca^2+^ affinity for TnC (*k*_1_) by 25% or 50%, which elicited greater pressure responses (Figure 6B; orange and green open circles). These simulated responses were driven solely by increased Ca^2+^ sensitivity with no contribution from cross-bridge responsiveness. To test the identifiability of this simple model, we sought to find a new set of parameters that could fit these simulated responses where only the parameters governing cross-bridge responsiveness were allowed to be modified (α, β, and *k*_d_); all other parameters were kept at control values. Using Matlab’s particle swarm optimizer, we found new parameter sets that could fit these sets of simulated responses to within 0.4% NRMSE (Figure 6B; orange and green lines). These parameter sets were identical to the control parameters except for increases in the values associated with the cross-bridge responsiveness parameters. We, therefore, have a model that can achieve similar fits to inotropic responses by increasing either the Ca^2+^ sensitivity or the cross-bridge responsiveness parameters. Hence, a simple model of Ca^2+^-myofilament dynamics is not identifiable for a data set that consists of Ca^2+^ inputs and force/pressure responses.

## 8 TnC conformation-based fluorophores

When Ca^2+^ binds to TnC, there are concomitant conformational changes to the proteins associated with the ‘activation’ of the thin filament (Gordon et al., 2000). Several groups have leveraged this phenomenon using targeted fluorescence probes to infer myofilament activation status (Hannon et al., 1992; Davis et al., 2007; Badr et al., 2016).

One such probe is 2-(4′-iodacetamidoanilino)naphthalene-6-sulfonic acid (IAANS; λ_ex_ 328 nm, λ_em_ 445 nm), which has a fluorescent intensity sensitive to the hydrophobicity of its local environment (Hannon et al., 1992). Ca^2+^ binding to TnC induces a conformational change that exposes a hydrophobic patch located in the B helix of TnC (Dong et al., 1999). Hence, an increase in fluorescent intensity should reflect the activation state of the thin filament. IAANS is introduced to permeabilised preparations by first removing the native TnC and reconstituting TnC labelled with IAANS (Johnson et al., 1980; Hannon et al., 1992). IAANS binds to wild-type TnC at Cys-35 and Cys-84, which are on opposite sides of the low-affinity regulatory Ca^2+^ binding site (Putkey et al., 1997). Due to possible modifications of TnC function caused by these binding locations (Putkey et al., 1997; Davis et al., 2007), Davis et al. (2007) developed a novel fluorescent TnC, where Thr-53 was mutated to Cys-53, which enabled accurate measurements of the rate of Ca^2+^ dissociation from the regulatory domain of TnC. However, IAANS-labelled TnC is currently limited to permeabilised samples.

Another recently developed conformation-based fluorophore uses fluorescence (or Förster) resonance energy transfer (FRET). FRET uses energy transfer between pairs of chromophores to measure intermolecular distance (Clegg, 1995). In cardiac applications, FRET-based sensors leverage the conformational change of TnC that occurs upon the binding of Mg^2+^ or Ca^2+^ to infer the extent of activation (Sevrieva et al., 2014; Badr et al., 2016). As with IAANS, FRET-based sensors require the replacement of endogenous TnC with a modified TnC that includes a pair of fluorophores. Initially, endogenous TnC was replaced in permeabilised samples (Badr et al., 2016), but a recent study replaced endogenous TnC in intact twitching cardiac preparations using a transgenic mouse model (Vetter et al., 2020). In each case, the modified cardiac preparations exhibited a reduced Ca^2+^ sensitivity (Badr et al., 2016; Vetter et al., 2020). There must also be concerns that, in a disease model, post-translational modifications arising from disease progression may differentially affect the modified and endogenous TnC proteins.

Though Vetter et al. (2020) mentions loading preparations with Fura-2 for Ca^2+^ measurement, the excitation and emission wavelengths of the donor fluorophore in the FRET pair (Clover; λ_ex_ 505 nm, λ_em_ 515 nm) are incompatible with this Ca^2+^-sensitive fluorophore (Fura-2; λ_ex_ 360 nm/380 nm, λ_em_ 510 nm). Using an alternative Ca^2+^ sensitive dyes that has an emission peak at shorter wavelengths, such as Indo−1 (λ_ex_ 335 nm, λ_em_ 405 nm/485 nm), or developing a FRET pair with a donor fluorophore operating at a longer wavelength may enable the simultaneous measurement of Ca^2+^ and TnC activation in intact cardiac preparations.

## 9 Closing remarks

*In vivo*, the heart is exposed to ever-changing conditions and demands. However, despite the number of methods discussed, there remains none capable of assessing the time course of sensitivity changes following a sudden change of conditions, for example, under post-rest potentiation with a sudden change of stimulus frequency (Bers et al., 1993) or in eliciting the slow force response with a sudden change of muscle length (Dowrick et al., 2019). The time course of myofilament Ca^2+^ sensitivity adaption presents a potential dimension to consider the deleterious impacts of cardiomyopathies. The limitations with existing methods for assessing transient sensitivity are derived from a need for more information on the distribution of reaction states. Force and Ca^2+^ measurements alone require a steady state in two different reactions (i.e., Ca^2+^-TnC and actin-myosin binding) to provide an insight into the sensitivity of cardiac muscle, which is not possible from physiologically twitching samples (Spurgeon et al., 1992). For sensitivity inference through a hybrid experimentation-modelling approach to become a valid approach, additional simultaneous state information is required. FRET-based TnC biosensors (Badr et al., 2016; Vetter et al., 2020) represent a step in this direction by providing a measure of the activation state of the thin filament, but they have yet to be used in conjunction with a cytosolic Ca^2+^ sensor, and they may overlook the contributions of post-translational modifications to the replaced myofilament proteins. The field is currently not equipped to assess transient myofilament Ca^2+^ sensitivity, but we propose that such a method will involve the use of simultaneously measured cytosolic Ca^2+^ and TnC activation in actively-twitching cardiac muscle preparations to identifiably parameterise a biophysical model.
